# Fidelity of a home-based pulmonary rehabilitation program in people with COPD referred from primary care

**DOI:** 10.1177/14799731241307247

**Published:** 2024-12-04

**Authors:** Simone Dal Corso, Anne E Holland, Johnson George, Michael J Abramson, Grant Russell, Nick Zwar, Billie Bonevski, Jaycie Perryman, Narelle S Cox

**Affiliations:** 1 Respiratory Research@Alfred, 2541 Monash University, Melbourne, VIC, Australia; 2 Institute for Breathing and Sleep, Melbourne, VIC, Australia; 3 Physiotherapy, 5392 Alfred Health, Melbourne, VIC, Australia; 4 School of Public Health and Preventive Medicine, 161667 Monash University, Melbourne, VIC, Australia; 5 Faculty of Health Sciences and Medicine, 104559 Bond University, Gold Coast, QLD, Australia; 6 College of Medicine and Public Health, 198094 Flinders University, Bedford Park, SA, Australia

**Keywords:** Pulmonary rehabilitation, telerehabilitation, chronic respiratory disease, primary care, exercise, telehealth

## Abstract

**Purpose:** Pulmonary rehabilitation (PR) is highly effective but underutilised. Pathways to home-based PR (HBPR) from general practice could improve utilisation, but program fidelity in this setting is unknown. This study aimed to explore the fidelity of HBPR in people referred from general practice. **Methods:** Secondary analysis of intervention-group data from two-arm cluster RCT (RADICALS-interdisciplinary intervention for people with COPD including smoking cessation support, home medicine reviews and 8-weeks HBPR). HBPR fidelity assessed by the extent to which exercise training was prescribed according to protocol. Completion of HBPR and contributing factors were determined. **Results:** 107 participants (68% of intervention group) were referred to HBPR, with *n* = 75 (70%) commencing the program (mean age 68 years, FEV_1_ 65% predicted, median mMRC 1). Aerobic training was prescribed according to protocol for 74% of participants in week one, and on average 89% of participants in weeks 2–8. Resistance training was prescribed according to protocol for 98% and 88% of participants (Week 1 and Weeks 2–8, respectively). Rehabilitation completers (*n* = 57, 76%) were 26 times more likely to have attended the Week 2 phone call (95% CI 2–352). Clinically meaningful improvements were achieved in health-related quality of life (SGRQ) and health status (CAT) following rehabilitation. **Conclusion:** PR program fidelity can be maintained when delivering HBPR to people with COPD referred directly from general practice. Early engagement with PR may be key to supporting rehabilitation completion.

## Background

Pulmonary rehabilitation is the primary non-pharmacological treatment intervention for people with chronic obstructive pulmonary disease (COPD). Recommended in international treatment guidelines,^1^ pulmonary rehabilitation is an evidence-based program of individually prescribed exercise training and education which improves symptoms and function, and reduces healthcare utilisation and hospital admissions.^2,3^ Despite its proven effectiveness, there are well documented patient and health-system barriers to pulmonary rehabilitation program access. Such barriers to rehabilitation participation include issues with travel and transport to attend programs, which are typically delivered at a centre^4^; insufficient numbers of programs, globally^5,6^; and low referral rates.^7^ To address these barriers, alternative models of rehabilitation delivery that provide pulmonary rehabilitation programs directly to people in their home have been demonstrated to be clinically^8^ and cost-effective.^9^ However, such programs still require referral to a specialist pulmonary rehabilitation service.

Finding new ways to offer pulmonary rehabilitation to people with COPD, outside of traditional centre-based pulmonary rehabilitation services, have been called for by international respiratory societies^10^ in an effort to improve program access and the proportion of individuals with chronic respiratory disease who go on to complete a pulmonary rehabilitation program. With more than 80% of people with COPD managed exclusively in primary care,^11^ integrating pathways to pulmonary rehabilitation within this setting could improve utilisation by creating a more seamless and accessible referral process.^12^ However, the fidelity of pulmonary rehabilitation programs delivered in the primary care setting, especially home-based ones, is unknown. Whether the core program components of pulmonary rehabilitation, specifically individually prescribed and progressed aerobic and resistance training and disease management education, can be delivered using a home-based service delivery model to individuals recruited directly from primary care has not been described in detail.

In this paper, we set out to describe the fidelity of a home-based pulmonary rehabilitation intervention delivered to people with COPD recruited directly from primary care as part of a larger clinical trial. The 8-week home-based rehabilitation model under evaluation has been previously described in detail.^13^ Briefly, the program comprises an initial home-visit with a physiotherapist to establish exercise goals, safety and undertake the first exercise training session. This is followed by seven once-weekly telephone calls, with rehabilitation staff trained in motivational interviewing, to progress exercise training, review symptoms and the home-exercise diary and facilitate self-management education. This home-based model of rehabilitation achieves similar clinical outcomes to traditional centre-based pulmonary rehabilitation^13^ and has been demonstrated to improve program access (94% of participants unable to access centre-based rehabilitation^14^); and completion (91% completion home-based vs 49% centre-based^13^). While intervention fidelity has been ascertained for programs delivered in clinical trials and via pulmonary rehabilitation services,^13,14^ whether the program model is maintained in a primary care setting has not been described.

The aims of this secondary analysis were to describe the fidelity of a home-based model of pulmonary rehabilitation delivered via primary care, and to characterise participant features associated with rehabilitation completion in this setting.

## Methods

### Study design

This paper reports a secondary analysis of intervention-group data from the RADICALS trial (Review of Airway Dysfunction and Interdisciplinary Community-based care of Adult Long-term Smokers), a two-arm, cluster randomised controlled trial involving 43 general practice (GP) clinics (ACTRN 12614001155684).^15^ RADICALS was an interdisciplinary intervention including smoking cessation support, home medicine reviews and home-based pulmonary rehabilitation.^15^ The present evaluation considers only the home-based pulmonary rehabilitation component for analysis.

### Participants

In the original trial,^15^ potential participants in each clinic were identified by a trained research assistant who contacted them via email or telephone. Individuals with confirmed COPD or undergoing COPD-specific treatment and who were at least 40 years old, had attended a minimum of two GP clinic visits in the previous year, with a smoking history of at least ten pack-years or those who had a documented diagnosis of COPD on clinic records or were being treated with COPD-specific medications were eligible for inclusion. After enrolment, the GP clinic provided the participant’s information—including spirometry results, dyspnoea scale (mMRC), and medical history—to our HBPR team. The HBPR team then assigned an experienced and trained physiotherapist for the home visit and relayed the participant’s details. Subsequently, the physiotherapist contacted the participant to schedule the visit at a mutually convenient day and time.

### Home-based pulmonary rehabilitation

Home-based pulmonary rehabilitation was delivered according to our previously established model for 8 weeks by a physiotherapist.^13^ In the initial home visit (Week 1), a trained physiotherapist set exercise goals for aerobic and resistance training, wrote a formal exercise prescription for the first week, supervised the first session, and instructed patients on using a diary to log their goals, planned exercises, and ascertained confidence levels. During the home visit, participants were provided with options for the days and times of the weekly phone calls scheduled for weeks 2–8. It was emphasized that participants should be available at their chosen day and time each week for 7 weeks, treating it akin to an outpatient medical appointment. If a participant did not answer the phone at the agreed time, the physiotherapist made follow-up contact on the same day and continued to make scheduled calls in the subsequent weeks, recording overall program attendance.

Throughout weeks 2–8, exercise objectives were progressively advanced during weekly phone calls conducted by physiotherapists trained in motivational interviewing, ensuring that the conversations were focused on building commitment and facilitating action.^16^ These phone calls served as a platform to discuss and establish weekly exercise goals, which participants then recorded in their diaries, noting specifics such as the duration and distance of walks and the types and number of resistance exercises to be aimed for during their unsupervised sessions. The physiotherapist conducting the weekly calls also documented all the details of the exercise program and educational sessions for the purposes of assessing the fidelity of the intervention. Aerobic exercise training was prescribed for at least 30 min comprising accessible activities, often involving walking, together with resistance training (upper and lower limbs) comprising functional activities and use of household items as weights for upper-limb training. Additionally, all participants were offered a selection of COPD self-care topics to choose from for weekly discussions, facilitating self-management education and goal setting. Moreover, the management of acute exacerbations and continued exercise participation were addressed with each participant at least once.

Further details about the home-based program can be found in the online supplementary material of Holland et al.^13^ or at the resource https://homebaserehab.net/.

### Outcome measures

Intervention fidelity was assessed by a pulmonary rehabilitation clinician independent of the trial. Clinician logs from intervention delivery were assessed to determine the extent to which aerobic and resistance training were prescribed according to protocol, and in keeping with recommended rehabilitation exercise dosing. Specifically, the fidelity components assessed were: (i) Aerobic training of at least 20 min/day (week 1) or at least 30 min/day (weeks 2–8) on at least 3 days in the week, (ii) At least 2 sessions per week of upper limb and lower limb resistance training; and (iii) Attainment of exercise training goals across program weeks 2–8. Fidelity components (i) and (ii) focussed on exercise prescription and progression, while component (iii) addressed participant engagement.

Education topics and health goals were also recorded. The proportion of individuals who completed rehabilitation, defined as undertaking at least 70% of the home-based pulmonary rehabilitation program,^13^ was recorded and contributing factors determined. Clinical outcome measures for the main trial (modified medical research council dyspnoea scale (mMRC), COPD assessment test (CAT), St Georges Respiratory Questionnaire (SGRQ) and Hospital Anxiety and Depression Scale (HADS) were collected at baseline and 6- and 12-months from baseline.^15^ Participant preference for timing of commencement of pulmonary rehabilitation meant that not all had completed the program by the time of 6-month assessment, so results were reported separately for those who did and did not complete pulmonary rehabilitation before this time point.

### Data analysis

Statistical analyses were carried out using Statistical Product and Service Solutions (SPSS version 28, IBM Corporation, Somers, USA). Only available data were used for the analyses. Data are presented as absolute values and percentages. For variables that followed a normal distribution, the mean and standard deviation were used, while for variables that followed a non-normal distribution, the median and interquartile range were used. For rehabilitation completers, unpaired t-tests or Mann-Whitney tests were used for comparison with non-completers based on the distribution, while the Chi-squared tests were used to compare categorical variables. *A priori* patient characteristics (age, sex, disease severity, and smoking status) and program features (attendance at Week 2 phone call) known to be associated with rehabilitation attendance were evaluated using Chi-squared or unpaired t-tests as appropriate. Variables demonstrating a significant relationship with program completion (*p* < .05) were included in a binary logistic regression analysis (backward stepwise). Differences in clinical outcomes over time were assessed according to distribution by Friedman’s test or repeated measures ANOVA with post hoc Bonferroni correction.

## Results

Of 107 participants (68% of intervention group) referred to home-based pulmonary rehabilitation, 75 (70%) commenced the program (*n* = 25 declined rehabilitation offer, *n* = 7 were unable to be contacted). Characteristics of individuals who chose to participate in home-based pulmonary rehabilitation and those who declined the offer of rehabilitation were similar, with the exception that those who accepted the offer of rehabilitation were about 5 years older [Table 1]. The majority (72%) of participants who commenced the program had mild-moderate COPD and the number of comorbidities was 4 (median, IQR 2 to 6).

**Table 1.** Baseline characteristics of participants who did and did not accept offer of home-based pulmonary rehabilitation.

Characteristics	Referral to home-based pulmonary rehabilitation	
	Accepted rehab (*n* = 75)	Declined rehab offer (*n* = 32)
Male *n* (%)	45 (60)	21 (66)
Age years	68 ± 10	63 ± 11*
BMI kg/m^2^	28 ± 6	27 ± 6
FVC % of predicted	87 ± 21	93 ± 17
FEV_1_ % of predicted	65 ± 21	72 ± 16
FER %	57 ± 14	60 ± 13
Smoking status		
Current *n* (%)	34 (57)	15 (60)
Quit *n* (%)	25 (42)	10 (40)
Never *n* (%)	1 (1)	0 (0)

Data are mean ± SD, unless indicated.

*p = .02.

Prescription of exercise training goals and goal attainment are detailed in Table 2. Aerobic training was prescribed according to the established criteria for 74% of participants during their week 1 home visit (≥20 min × 3/week), and for mean (SD) 89 (8)% of participants in weeks 2–8 (≥30 min × 3/week). Around half of participants attained aerobic training goals in weeks 2–8 (50 (5)%). Resistance training (≥2×/week) was prescribed according to protocol for 98% and 88 (5)% of participants in Week 1 and Weeks 2–8, respectively. Resistance training goals were achieved by 95 (3)% of participants in Weeks 2–8.

**Table 2.** Proportions of participants with exercise training prescribed according to the criteria and goal attainment over 8-week pulmonary rehabilitation program (*n* = 57).

	Week	Aerobic training		Resistance training	
		Prescription	Attainment	Prescription	Attainment
Home visit	1	74%	NA	98%	NA
MI calls	2	98%	53%	97%	98%
	3	95%	46%	95%	98%
	4	95%	53%	91%	96%
	5	91%	45%	83%	90%
	6	86%	46%	88%	98%
	7	83%	54%	84%	92%
	8	90%	56%	88%	92%
Mean ± SD	Weeks 2–8	89 ± 8	50 ± 5	91 ± 6	95 ± 3

Legend: MI: motivational interviewing; NA: not applicable.

Fifty-seven participants (76%) completed at least 70% of the rehabilitation program and were classified as rehabilitation completers (Table 3). On data review two additional rehabilitation completers were identified who were not reported in the main study findings. Individuals who completed rehabilitation had less severe disease (completers mean FEV_1_ 68% predicted vs non-completers 55% predicted), while attendance at the first scheduled rehabilitation phone call (week 2) was 26 times higher for rehabilitation completers than non-completers (Odds Ratio 26.2, 95% CI 2.0 to 352) (Table 4).

**Table 3.** Baseline characteristics and home-based pulmonary rehabilitation summary of program completers and non-completers.

	Home-based pulmonary rehabilitation program				
	Total (*n* = 75)	Completers (*n* = 57)	Non-completers (*n* = 18)	Mean or median difference (95% CI) completers vs non-completers	*p*-value
Baseline characteristics					
Male/female *n* (%)	45 (60)/30 (40)	36 (61) /23 (39)	9 (56)/7 (44)	NA	.73
Age	68 ± 10	68 ± 9	67 ± 12	1.4 (−4.0 to 6.9)	.61
BMI	28 ± 6	28 ± 6	26 ± 7	1.9 (−2.0 to 5.8)	.34
FVC %predicted	87 ± 21	91 ± 19	77 ± 23	14 (3.1 to 25.2)	.013
FEV_1_ %predicted	65 ± 21	68 ± 20	55 ± 22	13 (1.7 to 24.4)	.025
FVC/FEV_1_ ratio %	57 ± 13	57 ± 13	57 ± 16	0.08 (−7.8 to 7.9)	.98
Smoking status				NA	.83
Current *n* (%)	34 (45)	25 (44)	9 (50)		
Quit *n* (%)	25 (33)	19 (33)	6 (33)		
Never *n* (%)	1 (1)	1 (2)	0 (0)		
Clinical outcomes					
MMRC	1 (1−2)	1 (1−2)	1 (1−2)	0 (0 to 1)	.63
CAT	14 (8−20)	15.5 (8.25−20)	11 (8−20)	−2 (−7 to 2)	.29
SGRQ, total score	37 ± 18	39 ± 18	32 ± 18	−6.5 (−16.8 to 3.9)	.22
HADS-depression	5 (3–7)	5 (3–7)	4 (1–8)	−1 (−3 to 1)	.40
HADS-anxiety	5 (2–8)	5 (2–8)	7 (4–9)	1 (−1 to 4)	.31
Home visit and MI calls					
Home visit total time, min median (IQR)	105 (90–115)	105 (90–115)	97.5 (90–105)	0 (−15 to 0)	.323
Week 2 call attended *n* (%)	67 (89)	55 (96)	12 (67)	NA	.002
Number of total sessions median (IQR)	8 (6–8)	8 (7–8)	3 (2–5)	−4 (−5 to −3)	<.001
Total call time, min median (IQR)	145 (100–181)	163 (125–193)	41.5 (20.3–71.3)	−111 (−134 to −91)	<.001
Call time/session, min median (IQR)	23 (18–27)	24 (20–28)	19.5 (14.8–22.9)	−5.2 (−8.3 to −1.5)	.008
People who received education program *n* (%)	70 (93%)	57 (100)	13 (72)	NA	<.001
Education session/person median (IQR)	4 (2–5)	4 (3–6)	2 (0–3)	−3 (−4 to −2)	<.001

Data are expressed in mean ± SD unless indicated.

Legend: FVC: forced vital capacity; FEV_1_: forced expiratory volume in 1 s; IQR: interquartile range; MI: motivational interviewing; min: minutes; *n*: number; NA: not applicable.

**Table 4.** Factors associated with pulmonary rehabilitation program completion (*n* = 75) by multiple logistic regression.

	β (SE)	Exp (β)	95% CI	*p*	Nagelkerke R^2^ (%)
FEV_1_ % predicted	0.4 (0.02)	1.0	1.0 to 1.1	.014	
First MI call completed	3.3 (1.3)	26.2	1.9 to 352.3	.014	
Constant	−4.5 (1.8)	0.01	NA	.014	
					30

Abbreviations: FEV_1_: forced expiratory volume in 1 s; MI: motivational interviewing.

Seventy participants (93%) undertook at least one education session during the rehabilitation period (median 4 [2–5]), with strategies for managing an exacerbation (83%) and exercise for health/lung health (67%) the most frequently covered topics (Table 5).

**Table 5.** Education topics covered during MI calls.

Topic	*n* (%)
Managing exacerbations/chest infections	62 (83)
Ongoing exercise/exercise for lung health	50 (67)
Getting the right support/accessing other health professionals/community resources/when to go to GP	26 (35)
Correct use of medications/inhalers	26 (35)
Smoking cessation	22 (29)
Managing breathlessness/breathing strategies	21 (28)
Airway clearance	15 (20)
Pain management	10 (13)
Diet/Nutrition	10 (13)
Weight management/loss	10 (13)
Identifying exacerbations	4 (5)
Hydration	4 (5)
Sinus management	2 (3)
Depression	2 (3)
Understanding lung function tests results	2 (3)
Continence issue	2 (3)
Exercising safely/self-monitoring	2 (3)
Managing stress/anxiety	2 (3)
Weight increase	2 (3)
Managing constipation	1 (1)
Oxygen therapy	1 (1)
Coping with grief	1 (1)
Sleep hygiene	1 (1)
Falls and balance	1 (1)
Exercising in extreme heat	1 (1)
Exercise and acute pain	1 (1)
Managing palpitations	1 (1)
Understanding lung health	1 (1)
Exercise and diabetes	1 (1)

Clinically meaningful improvements in quality of life (SGRQ) and health status (CAT) were achieved following rehabilitation (Table 6). A significant improvement in reports of anxiety and depression was demonstrated at both 6- and 12-months from baseline for those who completed rehabilitation prior to 6-month follow-up (Table 6).

**Table 6.** Clinical outcomes over time for those who completed and did not complete the PR before the 6-month study assessment.

	PR undertaken before 6 mo assessment			PR undertaken after 6 mo assessment		
	Baseline (*n* = 45)	6-mo follow-up (*n* = 41)	12-mo follow-up (*n* = 39)	Baseline (*n* = 10)	6-mo follow-up (*n* = 7)	12-mo follow-up (*n* = 8)
mMRC	1 (1–2)	1 (1–2)	1 (1–2)	1.5 (1–3)	1 (0–2)	1.5 (0.25–2)
CAT	16 (9–20)	14 (8–17.5)	10 (6–18)	9.5 (5.5–21.5)	8 (5–11)	6.5 (3.75–11.75)
SGRQ total score, mean ± SD^a^	39 ± 17	35 ± 17	32 ± 18^a^	31 ± 18	34 ± 17	27 ± 16
HADS-depression	5 (3–8)	1 (0–5)^b^	1 (0–4)^d^	4.5 (2.5–5.25)	1 (0–1)	0 (0–1)
HADS-anxiety	5 (1.5–8.5)	3 (1.5–8.5)^c^	2 (0–4)^d^	4.5 (2.75–5)	0 (0–2)	0 (0–2.25)

LEGEND: Data expressed as median (IQR) unless otherwise specified.

^a^*p* = .03 versus baseline. ^b^*p* = .001 versus baseline. ^c^*p* = .035 versus baseline. ^d^*p* < .001 versus baseline.

^a^Repeated measures ANOVA with post hoc Bonferroni correction; all other comparisons Friedman’s test with a post hoc analysis using Wilcoxon signed-rank tests.

## Discussion

In people with COPD, the fidelity of a home-based pulmonary rehabilitation program can be maintained when delivered to individuals recruited directly from primary care. Core program components of individually prescribed and progressed aerobic and resistance training, as well as tailored education, were able to be provided to participants consistently across the 8-week program. The proportion of individuals who declined the offer of rehabilitation in this cohort was not different to previous reports,^17^ however the rate of rehabilitation completion was in keeping with outcomes of other home-based service delivery^14^ and exceeds that typically seen for outpatient pulmonary rehabilitation programs.^17^ Creating avenues for more individuals with COPD to complete a program of pulmonary rehabilitation is important, as rehabilitation completion is associated with a more than 50% reduction in healthcare utilisation^13^ and significant cost savings, irrespective of rehabilitation location.^9^

Engagement with the first scheduled program phone call as a key factor in participants proceeding to rehabilitation program completion is a novel finding. While patient specific factors, such as smoking status, disease severity and living situation, have been reported as both being facilitators and barriers to attendance at centre-based pulmonary rehabilitation,^18^ the timing of program engagement was unknown. In this model of home-based rehabilitation, the weekly phone call with the physiotherapist was scheduled at a set day and time, of the patients choosing, and framed as a regular appointment akin to visiting their general practitioner. In other outpatient settings, the failure to attend rate for outpatient neuropsychology in people with traumatic brain injury was nearly 50% when patients have not engaged with the pre-appointment triage phone call.^19^ Failure to attend first appointment was associated with an 11-fold greater risk of future non-attendance for paediatric outpatient clinics at a tertiary hospital.^20^ This suggests that concerted strategies to support rehabilitation engagement within the first weeks of the program may support ongoing participation and program completion.

The functional, symptomatic and well-being benefits of pulmonary rehabilitation are well-proven,^2,3^ yet rates of program referral, and subsequent uptake of referral, remain suboptimal.^7^ To date, very few strategies to improve referral to and uptake of pulmonary rehabilitation have been investigated.^21^ Although not the specific focus of this analysis – the uptake of home-based rehabilitation delivered directly from general practice seen here (70%) is in keeping with the proportion of people who attend an initial centre-based assessment (69%)^17^ and slightly greater than the proportion who typically go on to commence centre-based rehabilitation (59%).^17^ Recently, interventions and strategies to support referral to and uptake of pulmonary rehabilitation are being increasingly investigated, particularly for patients managed in primary care.^21^

In a systematic review of interventions, three studies (1 cluster RCT; 2 pre-post) of clinician and/or patient targeted education, with or without an individualised patient care plan, achieved pulmonary rehabilitation uptake rates of up to 25% more in the intervention group than the comparison group.^21^ Similarly, embedding a specialist cardiorespiratory physiotherapist into general practice to facilitate rehabilitation referral and support physical activity participation and self-management strategies resulted in just over one-third of participants having attended rehabilitation at 3-month follow-up.^22^ In contrast, a pre-rehabilitation introductory session saw fewer patients progress to rehabilitation assessment than usual care.^23^ The rehabilitation uptake rate of 70% seen in the current analysis may reflect the elimination of an additional assessment appointment, or the greater flexibility afforded to patients with a home-based intervention^24,25^ combined with the removal of common attendance barriers such as issues of travel and transport.^18^

A limitation of this report was that the location of intervention delivery precluded the assessment of exercise capacity immediately before or after rehabilitation, as is considered a core component of rehabilitation program delivery.^26^ While the fidelity of the model of home-based rehabilitation described here was maintained when delivered to individuals with COPD recruited from general practice, participants were more easily able to achieve training goals for resistance exercises. This may reflect the ease of performing functional exercises within the home environment, compared to an outdoors walking program. Delivery of rehabilitation programs in the home environment may need to consider a variety of aerobic training modalities to further support goal attainment. We were also unable to control for other interventions received during the study period (e.g. home medicines review) and as such we could not attribute changes in outcomes to the home-based rehabilitation intervention in isolation. Finally, our results should be interpreted cautiously as this study is a secondary analysis with a sample size determined by the original research.

Pulmonary rehabilitation program component fidelity, particularly as relates to exercise training prescription and progression and self-management education, can be maintained when delivering home-based rehabilitation to people with COPD directly from primary care. Early engagement with the rehabilitation intervention, specifically the first phone call consultation with the physiotherapist, appears key to supporting rehabilitation attendance and completion. To ensure model consistency with core pulmonary rehabilitation program requirements, procedures to enable evaluation of core assessment elements need to be implemented when delivering home-based rehabilitation to patients referred directly from primary care in the future.
